# G3 2024 Reviewer Index

**DOI:** 10.1093/g3journal/jkae261

**Published:** 2024-12-11

Below is a list of individuals who have reviewed manuscripts for G3: Genes | Genomes | Genetics during the preparation of Volume 13 (2023). The aim of G3: Genes | Genomes | Genetics is to communicate high-quality, well-described, useful research, across all areas of genetics and genomics. To succeed, we must recognize what meets our criteria for publication, making sure the presentation is accurate, unambiguous, and easily readable. For this, we depend heavily upon our reviewers, who perform this indispensable service without reward. The quality of the journal strongly reflects the quality of their efforts. We apologize to any reviewer whose name has been inadvertently omitted, and we are sincerely grateful to all.

Aarattuthodi, Suji

Aarts, Mark

Abbott, Wade

Abdelsalam, Nader

Ables, Elizabeth

Abouheif, Ehab

Abueg, Linelle

Acosta, Juan

Acquadro, Alberto

Adeleke, Matthew

Adiba, Sandrine

Aerts, Stein

Agami, Reuven

Aggarwal, Rashmi

Ahmad, Kami

Ahmed, Yashi

Ahn, Sang-Nag

Ahrendt, Steven

Ahringer, Julie

Akbari, Omar

Albà, Mar

Albertin, Caroline

Alexandre, Nicolas

Alioto, Tyler

Allard, Patrick

Allen, Mary

Allen, James

Allier, Antoine

Amini, Fatemeh

Amodeo, Amanda

Amundsen, Keenan

Andersen, Erik

Anderson, Matthew

Anderson, Jill

Andersson, Leif

Andorf, Carson

Anglin, Noelle

Antunes, Agostinho

Aoun, Nathalie

Apfeld, Javier

Arbeitman, Michelle

Arends, Danny

Arguello, J. Roman

Arick, Mark

Arimoto, Asuka

Armstrong, Ellie

Armstrong, Alissa

Ashcroft, Margaret

Ashikawa, Ikuo

Ashwell, Christopher

Aumailley, Monique

Awadalla, Phillip

Axelsson, Erik

Ayroles, Julien

Bachem, Christian

Bachvaroff, Tsvetan

Baer, Charles

Bageritz, Josephine

Bagnat, Michel

Bähler, Jürg

Bai, Jihong

Bai, Man

Baker, Scott

Bakonyi, Jozsef

Baldassarre, Daniel

Bálint, Miklós

Balint-Kurti, Peter

Bancic, Jon

Bandillo, Nonoy

Bannasch, Danika

Barahona, Sergio

Barbier, Estelle

Barghi, Neda

Barkoulas, Michalis

Barnes, Andrew

Barr, Maureen

Barr, Norman

Barrera-Redondo, Josué

Barrick, Jeffrey

Barrios, Arantza

Barshis, Dan

Barton, Nicholas

Basrai, Munira

Bastien, Catherine

Bateman, Jack

Baucom, Regina

Baulcombe, David

Baumann, Peter

Baxevanis, Andy

Baxter, Simon

Baxter, Simon

Bean, Tim

Beavis, William

Becraft, Philip

Beinart, Roxanne

Bell, Avery

Bellinger, M. Renee

Belmonte, Fernando

Belzile, François

Bender, Judith

Benner, Christopher

Benson, Mikael

Bentley, David

Berger, Dave

Bergey, Christina

Bergström, Tomas

Berman, Judith

Bernal, Moises

Bernardi, Giacomo

Bernardo, Rex

Bernards, Rene

Bernhardsson, Carolina

Bethke, Gerit

Betz, Regina

Beyer, Andreas

Bhattacharyya, Madan

Bickhart, Derek

Bi­en­hold, Christina

Bierman, Anandi

Bierne, Nicolas

Bilder, David

Billings, Timothy

Birchler, James

Bird, Kevin

Biscarini, Filippo

Bjørgen, Håvard

Blackmon, Heath

Blackwell, T.

Blevins, Todd

Bloom, Joshua

Blumenstiel, Justin

Bobadilla, Ana Clara

Boixel, Anne-Lise

Boland, Michael

Bollmann-Giolai, Anita

Bolon, Daniel

Bolterstein, Elyse

Bonini, Nancy

Bono, Lisa

Bontonou, Gwen

Boone, Charles

Borchers, Annette

Boria, Robert

Bosco, Giovanni

Botwright, Natasha

Bou, Marta

Bouchet, Sophie

Boukadida, Khouloud

Boukar, Ousmane

Boulant, Steeve

Boush, Mahnaz

Boutte, Cara

Bouvet, Jean-Marc

Bouwman, Aniek

Boyles, Richard

Braasch, Ingo

Bracewell, Ryan

Braden, Laura

Brault, Charlotte

Bravo, Gustavo

Bredy, Timothy

Brem, Rachel

Brewster, Rachel

Brilliant, Murray

Briscoe, James

Brito, Luiz

Broad, Gavin

Broderick, Nichole

Broders, Kirk

Brooks, Angela

Broquet, Thomas

Brown, C. Titus

Brown, Susan

Brown, Pat

Browning, Sharon

Bruick, Richard

Brumer, Harry

Brungardt, Jordan

Brusich, Douglas

Bruskin, Sergey

Bryan, Glenn

Buchon, Nicolas

Buckley, Reuben

Bult, Carol

Buntjer, Jaap

Buono, Russell

Burga, Alejandro

Burgess, Shawn

Burgueño, Juan

Bushley, Katherine

Byrne, Patrick

Caballero-Solares, Albert

Cachot, Jerome

Cai, Manjun

Calarco, John

Calic, Irina

Calipari, Erin

Calleja-Rodriguez, Ainhoa

Callister, Andrew

Campagna, Leonardo

Campbell, Polly

Campbell, Lesley

Campbell, Sharon

Campbell-Staton, Shane

Candy, John

Canizares, Joaquin

Cannon, Steven

Canovas, Angela

Carbone, Mary

Card, Daren

Cardenas, Thomas

Cárdenas, Leyla

Carlson, John

Carroll, Cassandra

Carson, Susan

Carvalho, Humberto

Casadei, Barbara

Casal, Margret

Castellano, David

Catchen, Julian

Catcheside, David

Cavendar-Bares, Jeannine

Cazares, Adrian

Cecere, Germano

Cerón Madrigal, Julián

Cervera, Maria

Chai, Yuan

Chakraborty, Mahul

Chalkia, Dmitra

Champer, Jackson

Chandra, Ambika

Chang, Ching-Ho

Chang, Michael

Chantret, Nathalie

Chappell, Joseph

Charlat, Sylvain

Charmantier, Anne

Charrier, Gregory

Chaudhary, Safee

Chaudhary, Bala

Chen, Qiuyue

Chen, Hang

Chen, Yun

Chen, Nan

Chen, Linxi

Chen, Chen-Hui

Chen, Hongquan

Chen, Yawei

Chen, Dahua

Chen, Fang

Chen, Chunyu

Chen, Hao

Chen, Yuanling

Chen, Huabang

Chen, Nansheng

Chen, Hua

Cheng, Hao

Cheng, Hans

Chevin, Luis-Miguel

Cheviron, Zachary

Chevre, Anne-Marie

Chevrette, Marc

Choi, Jae Young

Chopra, Surinder

Choudhary, Madhusudan

Chow, Clement

Chr Stenseth, Nils

Christensen, Alan

Chu, Ka Hou

Chung, J. Sook

Chung, SeYeon

Chuong, Cheng-Ming

Ciani, Elena

Cicconardi, Francesco

Cimadomo, Danilo

Civetta, Alberto

Clark, Emily

Clark, Andrew

Clarke, Robert

Claycomb, Julie

Close, Timothy

Clowney, E. Josephine

Cockett, Noelle

Coelho, Marco

Cogan, Noel

Colaiácovo, Monica

Collins, Kevin

Colome-Tatché, Maria

Conant, Gavin

Connallon, Tim

Conover, Justin

Conrad, Marcus

Conradt, Barbara

Coolon, Joseph

Cooper, Vaughn

Corak, Keo

Cortes Ciriano, Isidro

Costa e Silva, JoÃ£o

Costa-Neto, Germano

Covarrubias-Pazan, Giovanny

Cox, Rachel

Coyne, Clarice

Cram, Erin

Cripps, Richard

Crisp, Peter

Cristescu, Melania

Croll, Daniel

Crook, Nathan

Cros, David

Crown, Nicole

Cryan, Elli

Cryle, Max

Cuello, Clément

Cuevas, Jaime

Cullis, Brian

Cuomo, Christina

Curran, Sean

Cussiol, José Renato

Cutter, Asher

Cvekl, Ales

Cyert, Martha

D'Adamo, Patrizia

D'Amico-Willman, Katherine

Da Fonseca, Ruta

Dacks, Andrew

Dahl, Andy

Dainat, Jacques

Dandekar, Abhaya

Danzmann, Roy

Davey, Megan

David, Ingrid

Davies, Robert

Davik, Jahn

Day, Troy

De, Sandip

De Brito, Reinaldo

De Kloet, Siwo

De La Torre, Amanda

De Vega, Jose

Dearden, Peter

Debernardi, Juan

Degnan, Bernard

Demontis, Fabio

Demuth, Jeff

Dennis, Elizabeth

Denton, Robert

Denu, John

Derbyshire, Mark

Derks, Martijn

Deroche-Gamonet, Véronique

Derr, James

Des Marais, David

Deschenes, Robert

Dessimoz, Christophe

Devreotes, Peter

DeWitt, Noah

Diaz, Raul

Diaz-Garcia, Luis

DiCenzo, George

Dicke, Marcel

Dickinson, Daniel

Diepenbrock, Christine

Dierick, Herman

Dietrich, Fred

DiFazio, Stephen

Dillon, Marcus

Dirks, Alden

Dixon, Scott

Dodgson, Jerry

Dohlman, Henrik

Dornburg, Alex

Dorrington, Rosemary

Dorus, Steve

Doyle, Jeff

Doyle, Darragh

Drott, Mlton

Duan, Jingyue (Ellie)

Dudchenko, Olga

Dudley, Aimee

Duitama, Jorge

Dunham, Maitreya

Dupuis, Julian

Dupuis, Josée

Durland, Evan

Duronio, Robert

Dutta, Anik

Dvorak, Jan

Dyer, Kelly

Ecker, Joe

Edwards, Jeremy

Edwards, David

Edwards, Jode

Ehlke, Nancy

Eisen, Michael

Eizenga, Georgia

El-Hage, Nazira

El-Kassaby, Yousry

Elkon, Ran

Elliott, Kiona

Ellis, Robert P.

Ellison, Christopher

Elwood, Fiona

Emebiri, Livinus

Emeriewen, Ofere Francis

Emerson, J. J.

Ene, Iuliana

Engebrecht, JoAnne

Eskin, Eleazar

Espenshade, Peter

Evans, Matthew

Excoffier, Laurent

Fahrner, Jill

Famoso, Adam

Farman, Mark

Fast, Mark

Fasulo, Barbara

Faulk, Christopher

Fay, David

Fei, Zhangjun

Feigin, Charles

Fellers, John

Ferchaud, Anne-Laure

Fernandes, Carlos

Ferree, Patrick

Ferreira, Sandra

Ferrier, David Ellard Keith

Ferris, Martin

Feulner, Philine

Feyereisen, Rene

Fierst, Janna

Figueras, Antonio

Finley, Russell

Fishman, Lila

Folk, Petr

Fontaneto, Diego

Forrest, Douglas

Forstmeier, Wolfgang

Forsythe, Evan

Fortwendel, Jarrod

Fox, Thomas

Fraczek, Marc

Francis, Warren

Francisco, Carolina

Frand, Alison

Frandsen, Paul

Frank, Uri

Franken, Philipp

Fraser, Andrew

Freathy, Rachel

Freedman, Jonathan

Freeman, Krista

Freitag, Michael

Fresnedo-Ramírez, Jonathan

Friesen, Timothy

Friesen, Timothy

Friis, Guillermo

Fritsche Neto, Robeto

Fritsche-Neto, Roberto

Frohman, Michael

Frøkjær-Jensen, Christian

Furrow, Eva

Furrow, Robert

Furusawa, Go

Futai, Eugene

Gabaldón, Toni

Gabel, Christopher

Galaviz-Silva, Lucio

Gamperl, A.

Ganguly, Diep

Gao, Yuan

Gao, Li-zhi

Garber, Manuel

Garcia, Susana

Garcia, Antonio Augusto

Garcia, Antonio

García, L.

García-Estrada, Carlos

Garcia-Mas, Jordi

Garcia-Quintanilla, Meritxell

Garfield, Victoria

Gasch, Audrey

Gaynor, R.

Geck, Renee

Geisbrecht, Erika

Geiser, David

Gems, David

Gepts, Paul

Gerardo, Nicole

Gerke, Justin

Gerstein, Aleeza

Gewurz, Ben

Giet, Regis

Gilbert, Kimberly

Gilbey, John

Giolai, Michael

Girirajan, Santhosh

Gitler, Aaron

Glasscock, Edward

Glémin, Sylvain

Glenn, Anthony

Godfrey, R. Keating

Godinho, Raquel

Goff, Valorie

Goldammer, Tom

Golic, Kent

Gonen, Serap

Gonzalez, Josefa

Gonzalez-Gaitan, Marcos

Good, Jeffrey

Goodwin, Stephen

Gopalakrishnan, Shyam

Gore, Michael

Gorter, Florien

Gottschalk, Christopher

Gottschling, Dan

Goudet, Jérôme

Graham, Natalie

Grandjean, Frederic

Grant, Barth

Grassa, Christopher

Gratacap, Remi

Grayhack, Elizabeth

Graze, Rita

Green, James

Green, Alistair

Greenberg, Anthony

Greenspoon, Philip

Greenwald, Iva

Greenwood, Celia

Grenier, Cécile

Gresham, David

Gressler, Markus

Grewal, Savraj

Grigoriev, Igor

Groen, Simon

Groenen, Martien

Grover, Corrinne

Gruber, Jonathan

Grueneberg, Alexander

Grunwald, David

Gu, Xingyou

Guerreiro, Marco

Guiguen, Yann

Gumienny, Tina

Gundersen-Rindal, Dawn

Guo, Ximing

Gutenkunst, Ryan

Gutierrez, Benjamin

Gutierrez, Alejandro

Gutierrez, Alejandro

Gutjahr, Caroline

Guydosh, Nicholas

Halfon, Marc

Hall, Thomas

Hall, Sarah

Hallem, Elissa

Halloran, Mary

Hallsson, Jon

Halstead, Michelle

Hämälä, Tuomas

Hambleton, Sarah

Hamilton, Jill

Hamilton, John

Hampton, Randolph

Han, Chun

Han, Renzi

Hanna, Michael

Hansen, Ole

Hao, Weilong

Harbison, Susan

Hargreaves, Diana

Harrington, Lea

Harrison, Melissa

Harrison, Maria

Harrow, Jennifer

Hart, Anne

Harvey, Simon

Hassan, Karl

Hasson, Esteban

Hatfull, Graham

Hatmaker, E.

Havird, Justin

Hayward, Alexander

Hazlerigg, David

He, Feng

He, Jia-Qiang

Heard, Edith

Heckenhauer, Jacqueline

Hedgecock, Dennis

Hedrick, Philip

Hegarty, Matthew

Hegedus, Dwayne

Heikkinen, Marja

Held, Michael

Helker, Christian

Henderson, Ashley

Henderson, Ian

Hendry, Jason

Hengartner, Michael

Henk, Daniel

Herlyn, Holger

Heslot, Nicolas

Hibbett, David

Hickner, Paul

Hieter, Philip

Higgins, Paul

Hilgarth, Maik

Hill, Robert

Hiller, Michael

Hinomoto, Norihide

Hirannaiah, Pradeep

Hittinger, Chris

Hobert, Oliver

Holland, Mitchell

Holliday, Jason

Holmes, Scott

Hom, ERIK

Hopper, Anita

Hornos Carneiro, Maria Fernanda

Hosken, David

Hospital, Frederic

Hou, Jing

Hou, Linjing

Hou, Yali

Houston, Ross

Hu, Patrick

Hu, Jim

Huang, Wen

Huang, Weichun

Huang, Junli

Huber, Robert

Huber, Christian

Huberman, Lori

Hulse-Kemp, Amanda

Humphrey, Timothy

Hunt, Piper

Hussain, Waseem

Hussain, Fatima

Hyten, David

Ibrahim, Mohamed

Iino, Yuichi

Ikeda, Shigaku

Ingvarsson, Pär

Insua, Ana

Ioannidis, Alexander

Irazoqui, Javier

Isidro Sánchez, Julio

Isik, Fikret

Iversen, Martin

Iwata, Hiroyoshi

Jackson, Chris

Jackson, Daniel

Jacobsen, Steven

Jacobson, Allan

Jagadish, Krishna

Jagannathan, Sujatha

Jain, Kavita

Jamann, Tiffany

Jansen, Gert

Jansky, Shelley

Januschke, Jens

Janzen, Garrett

Jegga, Anil

Jenkins, Paul

Jenkins, Jerry

Jensen, Ingvill

Jensen-Seaman, Michael

Jeong, Soon-Chun

Jia, Zhenyu

Jia, Kailiang

Jiang, Xuejun

Jiang, Huaizhi

Jiang, Han

Jiang, Cen

Jiao, Renjie

Jieru, Wang

Jiggins, Chris

Jin, Long

Johansen, Kristen

Johansson, Anna

Johnson, Aaron

Johnson, Kevin

Johnson, Charles

Johnston, Mark

Johnston, J. Spencer

Johri, Parul

Jombart, Thibaut

Jones, Simon

Jones, Nick

Jones, Corbin

Jordan, Crispin

Jordan, David

Jose, Antony

Joshi, Vijay

Juliano, Celina

Junker, Jan Philipp

Kaandorp, Jaap

Kachroo, Aashiq

Kadosh, David

Kaelin, Chris

Kaeppler, Heidi

Kaeppler, Shawn

Kagey, Jacob

Kajiya-Kanegae, Hiromi

Kamfwa, Kelvin

Kamphorst, Jurre J.

Kanai, Muneyoshi

Kanca, Oguz

Kantanen, Juha

Kantorow, Marc

Kardos, Marty

Karp, Xantha

Kasbekar, Durgadas

Kashiwa, Takeshi

Kassis, Judith

Kastally, Chedly

Katz-Jaffe, Mandy

Kauffman, Kathryn

Kawahara, Akito

Keene, Alex

Keim, Paul

Kelada, Samir

Kelliher, Christina

Kellogg, Elizabeth

Kelly, Laura

Kelly, Tara

Kema, Gert

Kennedy, Peter

Kennell, Jennifer

Kenny, Nathan

Kern, Andrew

Kessner, Darren

Khatib, Hasan

Kijas, James

Kim, Bernard

Kim, Chang-Bae

Kimchi-Sarfaty, Chava

King, Elizabeth

King-Jones, Kirst

Kinney, Justin

Kitchen, Sheila

Klápště, Jaroslav

Klocko, Andrew

Koch, Evan

Koch, Sandra

Koch, Karen

Kohler, Annegret

Komiyama, Tomoyoshi

Kong, Fanjiang

Konkel, Zachary

Konopka, James

Konyves, Kalman

Koop, Ben

Koromila, Theodora

Kozik, Alexander

Krämer, Nicole

Krabbenhoft, Trevor

Krause, Margaret

Krause, Mirja

Kuhl, Joseph

Kuhn, Gustavo

Kukekova, Anna

Kulathinal, Rob

Kumar, Ritesh

Kumar, Arvind

Kupiec, Martin

Kurihara, Yukio

Kusch, Stefan

Kusmec, Aaron

Kwon, Taejoon

Kyriazis, Chris

Labbé, Simon

Labonte, Benoit

Labroo, Marlee

Lackey, Lela

Lackner, Laura

Laederach, Alain

Lai, Zhi-Chun

Lan, Xianyong

Landi, Maria Teresa

Langdon, Quinn

Lapègue, Sylvie

Lark, Karl

Larracuente, Amanda

Larsen, Peter

Larson, Greger

Larsson, Jan

Lassance, Jean-Marc

Latunde-Dada, Gladys O

Lauer, Edwin

Laumer, Christopher

Laurens, Pauwels

Lauterbur, M. Elise

Laverty, Kaitlin

Lavretsky, Philip

Law, Matthew

Lazetic, Vladimir

Le Rouzic, Arnaud

Leaché, Adam

Lebestky, Tim

Lecheta, Melise

Lee, Cheng-Ruei

Lee, Robert

Lee, Dong-Yup

Leeb, Tosso

Legarra, Andres

Legras, Jean-Luc

Lehner, Ben

Lehnert, Sarah

Lei, Li

Leitch, Andrew

Leite, Daniel

Lemaitre, Bruno

Lennon-Duménil, Ana-María

Lenormand, Thomas

Lenstra, Johannes

Leroux, Michel

Letsou, Anthea

Leulier, Francois

Levi, Amnon

Levin, Henry

Levine, Joel

Levine, Mia

Levy, Avraham

Lewis, James

Lewis, Zachary

Li, Fuhua

Li, Hongran

Li, Fei

Li, Yongxin

Li, Jibing

Li, Wenxuan

Li, Xianran

Li, Qi

Li, Wenli

Liang, Zhikai

Liang, Wanqi

Liang, Yan

Liao, Yi

Liao, Vivian

Libuda, Diana

Licata, Luana

Lieberman, Tami

Lieberman, Paul

Lin, Feng

Lin, Shuibin

Lin, Xinhua

Linck, Ethan

Linde, Celeste

Linder, Maurine

Lindsey, Amelia

Ling, Yinghui

Linscott, T. Mason

Lipka, Alexander

Lipps, Sarah

Lister, Ryan

Liu, Weiwei

Liu, Yie

Liu, Haoping

Liu, Yi

Liu, Yongbin

Liu, Dandan

Liu, Peng

Liu, Beidong

Liu, Sanzhen

Liu, Shuyu

Liu, Xiaochuan

Llopart, Ana

Lo, Sassoum

Logan, Cheryl A

Lohman, David

Lohmueller, Kirk

Loll-Krippleber, Raphael

London, Barry

Long, Manyuan

Long, Anthony

Looney, Brian

Lorenz, Aaron

Lovell, John

Lstibůrek, Milan

Lu, Lu

Lu, Qiongshi

Lucotte, Elise

Luigi-Sierra, Maria Gracia

Luke, Brian

Lummaa, Virpi

Luo, Ming-Cheng

Luschnig, Stefan

Lustig, Arthur

Lynch, Vincent

Lynch, Michael

Lyons, Leslie

Ma, Jian Feng

Ma, Jianxin

Macdonald, Stuart

Macias, Vanessa

MacKay, John

MacNeil, Lesley

Macqueen, Daniel

MacRae, Calum

Madsen, Ole

Mahan, Adam

Maina, Fanna

Mair, William

Makalowski, Wojciech

Malik, Harmit

Mallet, James

Malmberg, Russell

Manier, Mollie

Marcel, Thierry

Marchini, Jonathan

Marcotte, Edward

Marinkovich, M. Peter

Mariyappa, Daniel

Marks, Debbie

Maroilley, Tatiana

Marrano, Annarita

Martin, Chris

Martin, Andrew

Martin, Elena

Martin, Francis

Martin, Arnaud

Martin, Alicia

Martinez, Daniel

Martínez-Gómez, Pedro

Martinez-Reyes, Inmaculada

Marz, Manja

Masato, Nikaido

Masel, Joanna

Massa, Alicia

Massett, Michael

Mathers, Thomas

Mathers, Thomas

Mathieson, Iain

Matoo, Omera

Matsuoka, Makoto

Matus, David

Maurange, Cedric

Mauro, Vincent

McBride, Carolyn S

McCandlish, David

McCouch, Susan

McCusker, John

McDonald, Megan

McElhoe, Jennifer

McElroy, Kyle

McGinnis, Karen

McIntyre, Lauren

McKay, John

McKay, Stephanie

McLay, Todd

McNally, Kenneth

McVean, Gilean

McVey, Mitch

Meiklejohn, Colin

Meisel, Richard

Meller, Victoria

Menten, Bjorn

Merot, Claire

Merritt, Thomas

Mesnildrey, Sebastien

Messer, Philipp

Metzstein, Mark

Meyer, Rachel

Meyer, Barbara

Mhora, Terence

Michael, Todd

Michell, Craig

Mierisch, Jennifer

Migicovsky, Zoë

Miguel-Aliaga, Irene

Milbourne, Dan

Milkevych, Viktor

Miller, Danny

Miller, David

Ming, Guo-li

Minoarisoa, Rajerison

Mishra, Parul

Missaoui, Ali

Mitani, Shohei

Mitchell, Phil

Mitchell, Aaron

Mitchell-Olds, Thomas

Mochida, Keiichi

Mohan, Ryan

Mohr, Stephanie

Molin, Mikael

Momen, Mehdi

Monday, Philip

Mondo, Stephen

Montesinos-López, Osval

Montgomery, Tai

Moore, Karen

Moorjani, Priya

Mooser, Vincent

Moran, Rachel

Morgan, Bruce

Morota, Gota

Morris, Geoffrey

Morris, James

Morrow, Edward

Morschhäuser, Joachim

Moscou, Matthew

Mott, Richard

Moura de Sousa, Jorge

Muchero, Wellington

Mudge, Jonathan

Mueller, Lukas

Muir, Peter

Mujic, Alija

Mullens, Alex

Muñoz-Amatriaín, María

Murray, John

Murray, John

Murugesan, Manikandan

Mussmann, Steve

Muszynski, Michael

Mutalik, Vivek

Muyle, Aline

Myers, Chad

Mykles, Donald

Myles, Sean

Nagy, Laszlo

Nakano, Michiharu

Nakao, Hajime

Nakazawa, Takehito

Naya, Frank

Ndjiondjop, Marie-Noelle

Neale, David

Nechipurenko, Inna

Neiman, Maurine

Nelson, David

Nerbonne, Jeanne

Neufeld, Thomas

Neuhaus, Klaus

Nevado, Bruno

Neyhart, Jeffrey

Nguyen, Henry

Nicholas, Hannah

Nichols, Scott

Nichols, Krista

Normandeau, Eric

North, Trista

Novarina, Daniele

Novoa, Beatriz

Nowell, Reuben

Nuñez-Acuña, Gustavo

Nuñez-Lillo, Gerardo

Nyman, Tommi

Nystrom, Thomas

O'Grady, Patrick

O'Loghlen, Ana

O'Meara, Teresa

O'Rourke, Eyleen

Oakey, Helena

Ogura, Atsushi

Oh, Kevin

Oh, Dong-Ha

Olito, Colin

Oliveira, Carla

Olson, Eric

Østbye, Tone-Kari

Ostevik, Kate

Ottenburghs, Jente

Ou, Shujun

Ozerov, Mikhail

Paaby, Annalise

Padilla García, Nelida

Page, Antony

Paixão, Tiago

Palaiokostas, Christos

Palladino, Francesca

Pallares, Luisa

Palmer, Daniela

Palmer, Daniela

Palmer, Glen

Palmieri, Ferdinando

Palti, Yniv

Palu, Rebecca

Panchy, Nicholas

Pandey, Udai

Pantha, Pramod

Paps, Jordi

Parichy, David

Park, Yoonseong

Park, Hong Keun

Park, Julie

Park, Joong-Ki

Park, Taesung

Parkin, Isobel

Parsch, John

Paten, Benedict

Patil, Gunvant

Patil, Kiran

Patron, Nicola

Patterson, Larissa

Pavlicev, Mihaela

Pawlowski, Wojciech

Pè, Mario

Peace, Cameron

Peatmer, Eric

Peichel, Catherine

Pellecchia, Marco

Pellegrini, Matteo

Pellegrino, Mark

Pemberton, Josephine

Peñaloza, Carolina

Peona, Valentina

Pepper, Alan

Perez, Bruno

Pérez-Rodríguez, Paulino

Peris, David

Perlin, Michael

Peterlongo, Pierre

Peterson, Thomas

Petit, Marie-AgnÃ¨s

Petroni, Katia

Pettersson, Mats

Peyssonnaux, Carole

Pfeifer, Susanne

Pfennig, Aaron

Phadnis, Nitin

Phillips, Patrick

Piedrahita, Jorge

Piepho, Hans-Peter

Pierce, Jonathan

Piggott, Beverly

Pikaard, Craig

Pillus, Lorraine

Pinto, Brendan

Piovani, Laura

Pirani, Renata

Pires, J.

Piuri, Mariana

Plomion, Christophe

Plough, Louis

Podsiadlowski, Lars

Poland, Jesse

Porse, Bo

Port, Fillip

Poss, Kenneth

Possani, Lourival

Postma, Erik

Pot, David

Potter, Christopher

Poudel, Hari

Powell, Daniel

Powell, Owen

Pozzi, Luca

Prasad, Manoj

Pressoir, Gael

Převorovský, Martin

Pritchard, Victoria

Proctor, Robert

Prokop, Andreas

Pruett, Jessica

Pufall, Miles

Pukkila-worley, Read

Puzey, Joshua

Qin, Yuqi

Qu, Hao

Qu, Lujiang

Quek, Camelia

Quezada, Marianella

Varshney, Rajeev

Rabinow, Leonard

Raborn, R.

Raghavan, Chitra

Rahman, Atif

Ramasubramanian, Vishnu

Ranaghan, Matthew

Rand, Matthew

Rankin, Catharine

Rasheed, Awais

Rasmussen, Simon

Rasmussen, Jeffrey

Rastas, Pasi

Rastelli, Eugenio

Ratikainen, Irja Ida

Rau, Domenico

Reddy, Janardan

Redhai, Siamak

Redwan, Raimi

Reed, Laura

Reed, Robert

Regan, Tim

Rehbein, Peter

Reider, Leila

Reiner, David

Reinke, Aaron

Reinke, Valerie

Reis, Tânia

Remco, Bouckaert

Rep, Martijn

Rest, Joshua

Reuter, Max

Reynolds, Todd

Ribeiro, Carlso

Rice, Brian

Rideout, Elizabeth

Riedelsheimer, Christian

Rincent, Renaud

Riquet, Florentine

Rise, Matthew

Ritchie, Peter

Rivera-Colón, Angel

Roberts, Philip

Robertson, Hugh

Robinson, Jacqueline

Robledo, Diego

Roca, Alfred

Rocha, Isabel

Rochus, Christina

Rodrigues, Murillo

Rog, Ofer

Rogers, Jeff

Rogers, Rebekah

Rogers, Steve

Rogina, Blanka

Roh-Johnson, Minna

Rokyta, Darin

Rollmann, Stephanie

Romanov, Michael

Romero-Severson, Jeanne

Rosenthal, Joshua

Rosling, Anna

Ross-Ibarra, Jeffrey

Rossetto, Maurizio

Rothenberg, Ellen

Rottiers, Veerle

Rozas, Julio

Rudd, Jason

Ruden, Douglas

Runcie, Daniel

Runcie, Daniel

Rusche, Laura

Rusholme-Pilcher, Rachel

Rutkoski, Jessica

Ruvinsky, Ilya

Ruzicka, Filip

Rye, Morten

Saayman, Xanita

Sackton, Timothy

Sadhu, Meru

Saghai Maroof, M.

Saha, Malay

Sahl, Jason

Sakai, Toshiyuki

Sakhale, Sandeep

Salas-Fernandez, Maria

Salazar, Camilo

Sallam, Ahmad

Samakovlis, Christos

Sampaio, Jose

Sampaio, Ana

Samuel, Buck

Sanchez, Gustavo

Sankararaman, Sriram

Santos, Renato

Sapkota, Manoj

Sarkar, Abhishek

Sartor, Ryan

Sasaki, Atsuo

Satoh, Noriyuki

Saupe, Sven

Savage, Sharon

Savijoki, Kirsi

Scapim, Carlos

Schaap, Pauline

Schacherer, Joseph

Schattat, Martin

Schield, Drew

Schiffer, Philipp

Schindler, Daniel

Schmidt, Paul

Schmitz, Robert

Schneeberger, Korbinian

Schoenebeck, Jeffrey

Schraiber, Joshua

Schranz, M. Eric

Schreck, Nicholas

Schrioeder, Julian

Schuldiner, Maya

Schultz, Darrin

Schulze-Lefert, Paul

Schwemm, Michael

Scott, Maxwell

Scott, Maxwell

Scotti, Ivan

Seabury, Christopher

Sedlazeck, Fritz

Segvic-Bubic, Tanja

Seidel, Michael

Sekhon, Rajandeep

Sella, Guy

Sen, Śaunak

Şen, Alper

Seney, Marianne

Serrano-Saiz, Esther

Sert, Tijen

Sessitsch, Angela

Seternes, Ole Morten

Sethuraman, Arun

Seviiri, Mathias

Seydoux, Geraldine

Seymour, Danelle

Shaffer, John

Shani, Eilon

Shannon, Laura

Shao, Renfu

Shapira, Michael

Sharma, Prashant

Sharo, Andrew

Sheehan, Michael

Shen, Li

Shen, Kang

Shigenobu, Shuji

Shu, Qingyao

Shvartsman, Stanislav

Sidhu, Chandni

Siekmann, Arndt

Sieriebriennikov, Bogdan

Silva Pereira, Cristina

Sim, Sheina

Simakov, Oleg

Simianer, Henner

Simon, Sabrina

Sing, Tina

Singh, Varsha

Singson, Andrew

Sinha, Saurabh

Skern-Mauritzen, Rasmus

Slatkin, Montgomery

Slot, Jason

Slotkin, Richard

Small, Clayton

Smart, Lawrence

Smid, Eddy

Smith, Keriayn

Smith, Seth

Smith, Craig

Smith, Jacqueline

Soares Scaranto, Bianca Maria

Sockanathan, Shanthini

Sommer, Morten

Soneson, Charlotte

Song, Qijian

Song, Giltae

Sorenson, Michael

Sork, Victoria

Sorrells, Mark

Sosnowska, Katarzyna

Sourdille, Pierre

Sousa, Ana

Sousa Ferreira, Mafalda

Southall, Tony

Spana, Eric

Spatafora, Joseph

Spencer, Hamish

Spribille, Toby

Srinivasan, Jagan

Srivastava, Mansi

Stagljar, Igor

Stajich, Jason

Stam, Remco

Stam, Maike

Staton, Margaret

Steingrimsson, Eirikur

Stenhouse, Claire

Stensmyr, Marcus

Stergiopoulos, Ioannis

Sternberg, Paul

Stetter, Markus

Steuernagel, Burkhard

Stich, Benjamin

Stirling, Peter

Stockwell, Brent

Stoffel, Martin A.

Stone, John

Street, Nathaniel

Stribling, Dan

Su, Yuan

Su, Rui

Suh, Alexander

Sukumaran, Sivakumar

Sun, Yongfeng

Sun, Ren

Sundaram, Meera

Sundberg, John

Sung, Way

Sunkel, Claudio

Suter, Steven

Sutherland, Ben

Suttle, Curtis

Sverdlov, Serge

Swaminathan, Kankshita

Taagen, Ellie

Tabach, Yuval

Tabima, Javier

Tabolacci, Claudio

Takahashi, Aya

Takeuchi, Takeshi

Takou, Margarita

Talla, Venkat

Tamanoi, Fuyuhiko

Tamazian, Gaik

Tamazian, Gaik

Tan, Tianwei

Tan, Milton

Tandonnet, Sophie

Tanigawa, Yosuke

Tao, Yongfu

Taubert, Stefan

Taylor, Cormac

Taylor, Martin

Teeling, Hanno

Telford, Max

Tellier, Aurélien

Theissinger, Kathrin

Thilmony, Roger

Thines, Marco

Thompson, Andrew

Thompson, Christopher

Thompson, Addie

Thumma, Bala

Thyme, Summer

Tigano, Anna

Timp, Winston

Ting, Chau-Ti

Tixier-Boichard, Michele

Toews, Dave

Tokuriki, Nobuhiko

Toland, Amanda

Tolhurst, Daniel

Tollervey, David

Tollis, Marc

Tootle, Tina

Torkamaneh, Davoud

Torres, Tatiana

Trail, Frances

Tralamazza, Sabina

Tripodi, Pasquale

Tuck, Simon

Tunstrom, Kalle

Turner, Thomas

Tvedte, Eric

Udall, Joshua

Ueltzhöffer, Kai

Unckless, Robert

Underwood, Charles

Urbaneja, Alberto

Usui, Fumitake

Valdés, Juan Antonio

Valenzuela-Muñoz, Valentina

Van Bakel, Harm

Van de Peer, Yves

Van den Heuvel, Joost

Van den Heuvel, Sander

Van der Hoeven, Ransome

Van der Knaap, Esther

Van der Linden, Alexander

Van der Werf, Julius

Van Ghelder, Cyril

Van Gilst, Marc

Van Os, Hans

Vanderpool, Dan

Vaucheret, Herve

Vazquez, Ana

Vedanayagam, Jeffrey

Velasco, Vera

Velotta, Jonhathan

Vendrami, David

Venero Galanternik, Marina

Verde, Ignazio

Verhulst, Eveline

Verstrepen, Kevin

Vidal-Gadea, Andres

Vidales-Rodríguez, Luz Elena

Vieira, Maria Lucia

Vila, Roger

Vilgalys, Rytas

Visscher, Peter

Vivekanand, Pavithra

Voichek, Yoav

Volkan, Pelin

Voolstra, Christian

Voss-Fels, Kai

Wagner, Wolfgang

Wahls, Wayne

Walden, Kimberly

Walhout, Albertha

Walker, Amy

Walker, Graham

Wallace, Douglas

Walsh, Bruce

Wang, Huanwei

Wang, Adrienne

Wang, Qingzhi

Wang, Jeremy

Wang, Wei

Wang, Yue

Wang, Jinliang

Wang, Lu

Wang, Hao

Wang, Kan

Wang, Shao-Gang

Wang, Xiaowo

Wang, Mingshuang

Wang, Zhenlong

Wang, Huakun

Wang, Lizhi

Wang, Jiankang

Warburton, Marilyn

Ward, Robert

Wargelius, Anna

Warner, Ryan

Washburn, Jacob

Wassenegger, Michael

Waterhouse, Robert

Watson, Robert

Watts, Felicity

Wayne, Marta

Weber, Jennifer

Weber, Andreas P.M.

Webster, Timothy

Webster, Matthew

Wegrzyn, Jill

Weigel, Detlef

Weimer, Bart

Weissing, Franz

Wells, Michael

Welsh, Catherine

Welsh, Trista

Werner, Thomas

Wessells, Robert

Westerman, Kenneth

Westhues, Cathy

Wheat, Christopher

Wheeler, Travis

Whetten, Ross

Whitby, Matthew

White, Richard

White, Laura

White, Michael

Whitehall, Simon

Whitehead, Andrew

Whitworth, Cale

Wickner, Reed

Widlund, Per

Wigby, Stuart

Wilhelm, Jim

Willett, Kristine

Williams, Bryony

Williams, Ernest

Williams, Robert

Wilson, Alex

Wimmer, Valentin

Wimmer, Ernst

Windbichler, Nikolai

Wingfield, Brenda

Wittkopp, Patricia

Wolf, Jason

Wolfe, Benjamin

Wood, Daniel

Wood, Valerie

Wooldridge, Brock

Worthington, Margaret

Wozniak, Richard

Wright, Gabriel

Wright, Charlotte

Wright, Stephen

Wu, Xing

Wu, Di

Wu, Yi-Chun

Wu, Harry

Würschum, Tobias

Xia, Xuhua

Xia, Qi-Dong

Xiao, Lihua

Xiao, Andrew

Xing, Yi

Xu, Shuqing

Xu, Wu

Xu, Chunming

Xue, Ming

Yabe, Shiori

Yadav, Lav

Yaginuma, Toshinobu

Yamamoto, Yoshiharu

Yamasaki, Masanori

Yan, Huihuang

Yan, Yan

Yanai, Itai

Yang, Chin Jian

Yang, Qin

Yang, Emily

Yang, Jinliang

Yano, Masahiro

Yanowitz, Judith

Yao, Chi-Kuang

Yoder, Anne

Yongfeng, Zhou

Yoshioka, Yuki

Yu, Luyang

Yu, Jun

Yu, Jianming

Yuan, Hongmei

Yun, Cheol-Won

Yunliu, Zeng

Zahler, Alan

Zanders, Sarah

Zanella Zaccaron, Alex

Zdobnov, Evgeny

Zeeuwen, Patrick

Zeitlinger, Julia

Zelhof, Andrew

Zerfaoui, Mourad

Zhan, Xinli

Zhang, Zhichun

Zhang, Depeng

Zhang, Zhihai

Zhang, Yuanmeng

Zhang, Ting

Zhang, Gaotian

Zhang, Qingrun

Zhang, Yanling

Zhang, Huawei

Zhang, Qingbo

Zhang, Zong-Biao

Zhang, Yun

Zhang, Junke

Zhang, Guofan

Zhang, Zhiwu

Zhang, Ya-Ping

Zhao, Peng

Zhao, Meixia

Zhao, Yusheng

Zheng, Aiping

Zheng, Ye

Zhong, Xiuqin

Zhou, Yongfeng

Zhou, Zheng

Zhou, Zhaolan

Zhou, Yingbing

Zhou, Baoliang

Zhou, Jun

Zimin, Aleksey

Zinovyeva, Anna

Ziolkowski, Piotr

Zirin, Jonathan

Zotta, Teresa

Zou, Jun

Zou, Fei

Zuo, Jinhua

Zyprian, Eva

